# The impact of immunotherapy on the survival of pancreatic adenocarcinoma patients who received definitive surgery of the pancreatic tumor: a retrospective analysis of the National Cancer Database

**DOI:** 10.1186/s13014-020-01569-5

**Published:** 2020-06-03

**Authors:** Saber Amin, Michael Baine, Jane Meza, Morshed Alam, Chi Lin

**Affiliations:** 1 Department of Radiation Oncology, University of Nebraska Medical Center, 986861 Nebraska Medical Center, Omaha, NE 68198-6861 USA; 2 Department of Biostatistics, College of Public Health, University of Nebraska Medical Center, Omaha, USA

## Abstract

**Background:**

Immunotherapy has paved the way for new therapeutic opportunities in cancer but has failed to show any efficacy in Pancreatic Adenocarcinoma (PDAC), and its therapeutic role remains unclear. The objective of this study is to examine the impact of immunotherapy in combination with chemotherapy, RT, and chemoradiation on the overall survival (OS) of PDAC patients who received definitive surgery of the tumor using the National Cancer Database (NCDB).

**Methods:**

Patients with PDAC who received definitive surgery of the pancreatic tumor and were diagnosed between 2004 and 2016 from the NCDB were identified. Cox proportional hazard analysis was used to assess the survival difference between patients who received chemotherapy plus immunotherapy and chemoradiation therapy plus immunotherapy and their counterparts who only receive these treatments without immunotherapy. The multivariable analysis was adjusted for age of diagnosis, race, sex, place of living, income, education, treatment facility type, insurance status, year of diagnosis, and treatment types such as chemotherapy and radiation therapy.

**Results:**

In total, 63,154 PDAC patients who received definitive surgery of the tumor were included in the analysis. Among the 63,154 patients, 636 (1.01%) received immunotherapy. Among patients who received chemotherapy (21,355), and chemoradiation (21,875), 157/21,355 (0.74%) received chemotherapy plus immunotherapy, and 451/21,875 (2.06%) received chemoradiation plus immunotherapy. Patients who received chemoradiation plus immunotherapy had significantly improved median OS compared to patients who only received chemoradiation with an absolute median OS benefit of 5.7 [29.31 vs. 23.66, *p* < 0.0001] months. In the multivariable analysis, patients who received immunotherapy had significantly improved OS compared to patients who did not receive immunotherapy (HR: 0.900; CI: 0.814–0.995; *P* < 0.039). Patients who received chemoradiation plus immunotherapy had significantly improved OS compared to their counterparts who only received chemoradiation without immunotherapy (HR: 0.852 CI: 0.757–0.958; *P* < 0.008).

**Conclusions:**

In this study, the addition of immunotherapy to chemoradiation therapy was associated with significantly improved OS in PDAC patients who received definitive surgery. The study warrants further future clinical trials of immunotherapy in PDAC.

## Background

Pancreatic adenocarcinoma (PDAC) is the 7th leading cause of global cancer deaths and the third leading cause of cancer deaths in the United States [1]. In 2019, there were an estimated 56,000 new cases of PDAC and 450,000 deaths [2]. It is predicted that PDAC will become the second leading cause of cancer deaths by 2030, after lung cancer [3]. There are no early detection tests, and most patients with localized disease have no recognizable symptoms or signs, and therefore, most PDAC patients are diagnosed after their cancer has metastasized to other organs [4]. The five-year survival rate for all stages remains at 5% and has not changed in the last 30 years [2].

Surgery is the only curative treatment, but unfortunately, only 15–20% of patients present with cancer that is amenable to resection [5]. Despite significant improvement in surgical techniques, the five-year survival rate after resection remains at 10–20% with a median survival of 24 months [5, 6]. A Locoregional and distant recurrence rate of up to 80% after surgery is reported, which is likely secondary to the presence of occult micrometastatic disease at the time of resection [7, 8]. The majority of locoregional or distant recurrence occurs within two years after resection [7–9]. The potential of PDAC for early metastases have convinced scientists to hypothesize that PDAC is a systemic disease at the time of diagnosis, even when there is no radiographic evidence of distant metastases [6].

Chemotherapy and/or chemoradiation have been combined with surgery to improve disease control and survival. Unfortunately, the outcomes of combined treatment are still not very promising. Therefore, there is a desperate need for more effective systemic therapy that could be combined with the current standard treatment to improve the overall survival (OS) of the resectable PDAC patients. Strategies of combining novel treatments such as immunotherapy with surgery have been proposed and could provide a potential successful curative option for PDAC patients. After making first inroads in cancer in the setting of metastatic melanoma in 2011, immunotherapy has now been approved for various malignancies [10, 11].

Immunotherapy is not approved for PDAC, and despite its lack of efficacy in the initial trials of mono immunotherapy, it has still been used primarily in the metastatic setting as a last-ditch effort following the failure of currently FDA approved therapies [12–15]. However, new evidence indicates that immunotherapy could be effective and useful in patients with localized disease who have a high risk of micrometastases a critical hallmark of PDAC [16–20].

Immunotherapy may be useful in PDAC patients who receive definitive surgery if it is combined with other treatments such as chemotherapy and radiation therapy (RT). Preclinical and clinical evidence demonstrates that immunotherapy can have synergistic interaction with chemotherapy and RT as they increase tumor-specific T cell infiltration, decrease Treg cells, and suppress Myeloid-derived suppressor cells (MDSCs) [19, 21]. In preclinical studies of PDAC, immunotherapy has elicited tumor regression and improved survival when used in combination with other treatments of cancer, especially chemotherapy [22, 23]. The aim of the current study is to investigate the impact of immunotherapy combined with chemotherapy and chemoradiation on the overall survival of PDAC patients who received definitive surgery of PDAC using the National Cancer Database (NCDB).

## Methods

### Data source

The data for this study was extracted from the National Cancer Database (NCDB), which is a joint program of the Commission on Cancer of the American College of Surgeons and the American Cancer Society. It captures 70% or more of newly diagnosed malignancies in the United States annually. This study was exempt from the Institutional Review Board (IRB) because the de-identified file of the NCDB data was used.

### Study population

The study included patients age 18 or older who were diagnosed with PADC between 2004 and 2016 and received definitive surgery of the tumor. Only patients who were diagnosed with PDAC were included using the ICD-O-3 histology codes of 8000, 8010, 8020–8022, 8140, 8141, 8211, 8230, 8500, 8521, 8050, 8260, 8441, 8450, 8453, 8470–8473, 8480, 8481, 8503,8250,8440, 8560. The surgical site-specific code was used to identify patients with definitive surgery of the tumor. Patients who were missing information about RT, chemotherapy, and immunotherapy were excluded. We also excluded patients with the M1 stage and those with unknown or missing information about other covariates in the adjusted multivariable analysis.

### End points

The primary outcome of the current study was the OS of the patients, which was calculated from the date of diagnosis to the date of death. Patients who were alive or lost to follow up were censored.

### Explanatory variables

The main predictors of OS in this study were immunotherapy, immunotherapy combined with chemotherapy, and immunotherapy combined with chemoradiation. The age of diagnosis, gender, race, urban and rural living status, income, education, treatment facility type, comorbidity score, insurance status, year of diagnosis, and receipt of chemotherapy, RT, and immunotherapy were other explanatory variables used in the analysis.

### Statistical analyses

Descriptive statistics were reported for categorical and continuous variables. Multivariable logistic analysis was used to identify the predictors of receiving immunotherapy and reported the odds ratio as a measure of association with the probability of receiving immunotherapy. The OS rates were determined using the Kaplan–Meier method and were compared between groups using log-rank statistics. Survival time was measured in months from the date of diagnosis to the date of death. Cox proportional hazards model was used to determine the significant predictors of OS and estimate the hazard ratio of death as well as its 95% confidence interval (CI). The multivariable analysis was adjusted for the age of diagnosis, gender, race, urban and rural living status, income, education, treatment facility type, comorbidity score, insurance status, year of diagnosis, and receipt of chemotherapy and RT. All the tests used in this study were two sided and *P* values < 0.05 was considered as statistical significance. All statistical analyses were carried out using SAS 9.4. Cary, NC: SAS Institute Inc.

## Results

In total, 63,154 patients diagnosed with PDAC between 2004 and 2016 who received definitive surgery of the tumor were included in the analysis. Among the 63,154 patients, 636 (1.01%) received immunotherapy. Among patients who received chemotherapy (21,355), and chemoradiation (21,875), 157/21,355 (0.74%) received chemotherapy plus immunotherapy, and 451/21,875 (2.06%) received chemoradiation plus immunotherapy. In the multivariable logistic analysis, older age, female sex, Black race, Charlson/Deyo Score of 1 and 2, treatment at a community hospital, being less educated, diagnosed before 2011, not receiving chemotherapy, and not receiving RT were significantly less likely to receive immunotherapy. The odds ratio of these factors is provided in Table 1.

**Table 1:** Multivariable logistic analysis of the predictor of immunotherapy in PDAC patients who received definitive surgery of the pancreatic tumor

Variable		Immunotherapy 636 (1.01%)	No Immunotherapy 62,518 (98.99%)	Total 63,154	Odds Ratio	95% CI	P
Age at diagnosis, Median (range)		62.00 (29–90)	67.00 (18–90)	63,154	0.973	0.965–0.981	< 0.0001
Sex	Male	352 (55.35)	31,719 (50.74)	32,071 (50.78)	1	Reference	
	Female	284 (44.65)	30,799 (49.26)	31,083 (49.22)	0.844	0.715–0.997	0.046
Race	White	574 (92.13)	53,761 (86.84)	54,335 (86.89)	1	Reference	
	Black	28 (4.49)	5982 (9.66)	6010 (9.61)	0.479	0.323–0.710	0.0003
	Other	21 (3.37)	21,68 (3.50)	2189 (3.50)	0.787	0.483–1.283	0.338
	Unknown	13	607	620			
Education	> = 13% HG	167 (26.47)	24,941 (40.05)	25,108 (39.91)	0.649	0.538–0.784	0.0001
	< 13%	464 (73.53)	37,336 (59.95)	37,800 (60.09)	1	Reference	
	Unknown	5	241	246			
Income	> = $35,000	459 (72.74)	38,308 (61.54)	38,767 (61.65)	1	Reference	
	< 35,000	172 (27.26)	23,944 (38.46)	24,116 (38.35)		NS	0.160
	Unknown	5	266	271			
Place of Living	Urban	604 (99.02)	59,667 (98.11)	60,271 (98.12)	1	Reference	
	Rural	6 (0.98)	1150 (1.89)	1156 (1.88)	0.414	0.154–1.114	0.081
	Unknown	26	1701	1727			
Hospital Type	Academic	505 (80.41)	34,074 (55.04)	34,579 (55.30)	1	Reference	
	Community	123 (19.59)	27,831 (44.96)	27,954 (44.70)	0.261	0.212–0.322	0.0001
	Unknown	8	613	621			
Insurance Status	Insured	623 (98.89)	60,145 (97.73)	60,768 (97.74)	1	Reference	
	Not insured	7 (1.11)	1399 (2.27)	1406 (2.26)	0.503	0.237–1.069	0.074
	Unknown	6	974	980			
Charlson/Deyo Score	0	486 (76.42)	40,852 (65.34)	41,338 (65.46)	1	Reference	
	1	125 (19.65)	16,270 (26.02)	16,395 (25.96)	0.728	0.591–0.896	0.003
	> = 2	25 (3.93)	5396 (8.63)	5421 (8.58)	0.519	0.340–0.792	0.002
Chemotherapy	Yes	608 (95.60)	42,622 (68.18)	43,230 (68.65)	1	Reference	
	No	28 (4.40)	19,896 (31.82)	19,924 (31.55)	0.209	0.138–0.316	0.0001
Radiation Therapy	Yes	459 (72.17)	22,068 (35.30)	22,527 (35.67)	1	Reference	
	No	177 (27.83)	40,450 (64.70)	40,627 (64.33)	0.350	0.289–0.425	< 0.0001
Year of Diagnosis	2004–2010	330 (51.89)	27,978 (44.75)	28,308 (44.82)	1.268	1.073–1.499	< 0.005
	2011–2016	306 (48.11.)	34,540 (55.25)	34,846 (55.18)	1	Reference	

When we excluded insurance status and place of living the results were the same; therefore, we included them in the multivariable analysis

**Fig. 1:**
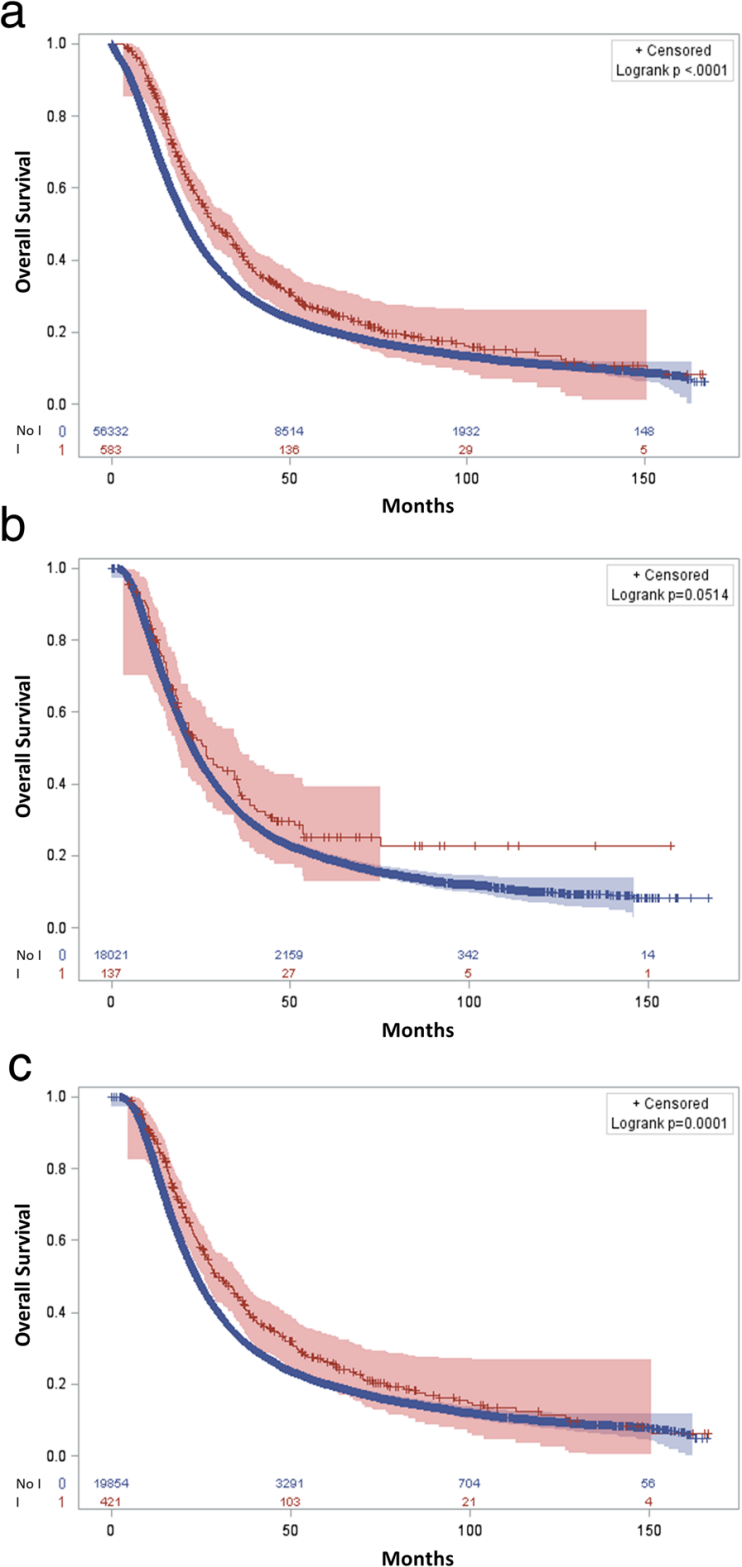
Overall survival with (red) or without (blue) immunotherapy for (**a**) all patients; (**b**) patients who received chemotherapy; (**c**) patients who received chemoradiation therapy;

PDAC patients who received immunotherapy had significantly improved median overall survival OS with an absolute median OS benefit of 7.1 [28.45 vs. 21.36; *p* < 0.0001] (Fig. 1a) months compared to their counterparts without immunotherapy. Patients who received chemoradiation plus immunotherapy had significantly improved median OS compared to patients who only received chemoradiation with an absolute median OS benefit of 5.7 [29.31 vs. 23.66; *p* < 0.0001] months (Fig. 1c). There was no significant difference in the median OS of patients who received chemotherapy plus immunotherapy and those who only received chemotherapy [26.28 vs. 22.70; *p* < 0.051] months (Fig. 1b). However, the extended plateaued or nearly a flat line at the end of the KM curve is indicative of the long-lasting immunity or cure from cancer, which is only seen in patients who received both chemotherapy and immunotherapy.

In the univariate Cox Proportional analysis (Table 2), patients who received immunotherapy had significantly improved OS compared to their counterparts without immunotherapy (HR: 0.773, CI: 0.702–0.850; *P* < 0.0001). Patients receiving chemoradiation plus immunotherapy had significantly improved OS compared to chemoradiation alone (HR: 0.804, CI: 0.718–0.899; *p* < 0.0001) (Table 3). In the univariate Cox Proportional analysis, patients who received chemotherapy plus immunotherapy did not notice significantly improved OS compared to their counterparts (HR: 0.818, CI: 0.668–1.002; *p* < 0.052) (Table 3).

**Table 2:** Univariable and multivariable Cox analysis of PDAC patients who received definitive surgery of the pancreatic tumor

Variable		Univariable analysis		Multivariable analysis	
		Hazard Ratio (95% CI)	P	Hazard Ratio (95% CI)	P
Age at diagnosis (continuous)		1.014 (1.013–1.015)	< 0.0001	1.012 (1.011–1.013)	< 0.0001
Sex	Male	Reference		Reference	
	Female	0.937 (0.919–0.955)	< 0.0001	0.925 (0.907–0.943)	< 0.0001
Race	White	Reference		Reference	
	Black	1.020 (0.988–1.054)	< 0.226	1.029 (0.994–1.064)	< 0.102
	non-white non-black	0.819 (0.774–0.867)	< 0.0001	0.856 (0.807–0.908)	< 0.0001
Education	> = 13% HG	1.119 (1.097–1.141)	< 0.0001	1.071 (1.045–1.096)	< 0.0001
	< 13% HG	Reference		Reference	
Income	> = $35,000	Reference		Reference	
	<$35,000	1.145 (1.123–1.167)	< 0.0001	1.091 (1.065–1.117)	< 0.0001
Place of Living	Urban	Reference		Reference	
	Rural	1.140 (1.064–1.222)	< 0.0002	NS	0.150
Hospital Type	Academic	Reference		Reference	
	Community	1.199 (1.176–1.222)	< 0.0001	1.198 (1.174–1.222)	< 0.0001
Insurance Status	Insured	Reference		Reference	
	Not insured	0.964 (0.903–1.028)	0.196	1.081 (1.011–1.156)	< 0.024
Charlson/Deyo Score	0	Reference		Reference	
	1	1.099 (1.075–1.124)	< 0.0001	1.061 (1.038–1.086)	< 0.0001
	> = 2	1.302 (1.258–1.348)	< 0.0001	1.232 (1.189–1.276)	< 0.0001
Year of Diagnosis	2004–2010	1.156 (1.134–1.179)	0.0001	1.155 (1.132–1.179)	0.0001
	2011–2016	Reference		Reference	
Chemotherapy	Yes	Reference		Reference	
	No	1.217 (1.192–1.242)	< 0.0001	1.137 (1.109–1.165)	< 0.0001
Radiation Therapy	Yes	Reference		Reference	
	No	1.117 (1.095–1.139)	< 0.0001	1.032 (1.008–1.057)	< 0.008
Immunotherapy	Yes	0.773 (0.702–0.850)		0.900 (0.814–0.995)	
	No	reference	< 0.0001	reference	< 0.039

**Table 3:** Univariate and multivariate Cox analysis of Combining Immunotherapy with Chemotherapy and chemoradiation therapy in PDAC patients who received definitive surgery of the pancreatic tumor

Variable		N (%)	Univariable analysis		Multivariable analysis	
			Hazard Ratio (95% CI)	P	Hazard Ratio (95% CI)	P
Chemo and immunotherapy combination	Chemotherapy Only	21,198 (99.26%)	Reference		Reference	
	Chemo + Immunotherapy	157 (0.74%)	0.818 (0.668–1.002)	< 0.052	NS	0.435
Chemoradiation and immunotherapy combination	Chemoradiation Only	21,424 (97.94%)	Reference		Reference	
	Chemoradiation + Immunotherapy	452 (2.06%)	0.804 (0.718–0.899)	< 0.0001	0.852 (0.757–0.958)	0.008

Two different models were developed for the multivariable analysis of Table 3 because these variables were mutually exclusive

In the multivariable Cox Proportional analysis, immunotherapy, female gender, and non-white non-black race were associated with significantly improved OS, while older age, Black race, treatment at community hospital, low income, low education, not receiving chemotherapy or RT, not having insurance, Charlson/Deyo of one and two, and diagnosis before 2011 were associated with significantly decreased OS (Table 2). The multivariable analysis was adjusted for age of diagnosis, race, sex, place of living, income, education, hospital type, insurance status, year of diagnosis, and Charlson/Deyo score. Patients who received immunotherapy had significantly improved OS compared to patients who did not receive immunotherapy (HR: 0.900; CI: 0.814–0.995; *P* < 0.039) (Table 2). Patients who received chemoradiation plus immunotherapy had significantly improved OS compared to their counterparts who only received chemoradiation without immunotherapy (HR: 0.852 CI: 0.757–0.958; *P* < 0.008) (Table 3). The 1-year and 2-year survival rates were 88 and 60% for chemoradiation plus immunotherapy patients compared to 81 and 49% in patients who only received chemoradiation (data not shown). Chemotherapy plus immunotherapy was not associated with significantly improved OS (Table 3).

## Discussion

Using the NCDB, this study examined the impact of immunotherapy in combination with chemotherapy and chemoradiation on the OS of PDAC patients who received definitive surgery of the tumor. Chemoradiation but not chemotherapy alone plus immunotherapy was associated with significantly improved OS in the univariate and multivariable Cox Proportional analysis adjusted for age of diagnosis, gender, race, income, education treatment facility type, Charlson/Deyo score, place of living, year of diagnosis, and insurance status.

The tumor microenvironment of PDAC is non-immunogenic and immunosuppressive [19]. Pancreatic cancer itself induces local and systemic immune dysfunction or immunosuppression to avoid being recognized and attacked by effector immune cells [24, 25]. The tumor cells use mechanisms such as the up-regulation of immune checkpoint signaling program (PD-L1, CTLA-4), the blockage of co-stimulation to activate T cells, and the recruitment of MDSCs, and tumor-associated macrophages to achieve immune suppression [26–28]. The tumor microenvironment reflects a lack of tumor-infiltrating lymphocytes and dendritic cells and plenty of suppressor T cells [29, 30]. Various rational combination treatment strategies have been proposed to overcome the resistance of PDAC to immunotherapy, including the combination of immunotherapies with chemotherapy and chemoradiation [31, 32].

Chemoradiation can work synergistically with immunotherapy and improve OS compared to chemoradiation alone. Chemotherapy and RT cause the release of neoantigens and upregulation of inflammatory cytokines, which promote the presentation of the neoantigens in the tumor microenvironment and thereby increase the immunogenicity of the tumor cells making them better targets for immunotherapy [33–35].

Checkpoint blockade immunotherapy has resulted in impressive responses in the metastatic setting of various tumors and, more recently, has been tested in the adjuvant setting after surgery [29, 30]. FDA has approved a couple of checkpoint inhibitors for adjuvant use in advanced melanoma, cervical cancer, bladder cancer, and renal cancer [30, 36]. Various types of immunotherapies, including checkpoint inhibitors and vaccines therapies in combination with chemotherapy and chemoradiation, have been studied in early-stage and metastatic PDAC but have not led to the FDA approval of immunotherapy for pancreatic cancer [37]. The use of immunotherapy in neoadjuvant or adjuvant setting combined with chemoradiation in PDAC has been limited. Some clinical trials studying the efficacy of immunotherapy in resectable PDAC combined with chemoradiation therapy have shown positive response and measurable activity [38–41]. The immunotherapy group represents only 1.01% of patients who received definitive surgery of the pancreatic tumor, indicating that this is a very highly select group of patients, and many of these patients might have been enrolled in clinical trials. Immunotherapy is not a standard-of-care treatment in pancreatic cancer outside of clinical trials. However, some patients are receiving Immunotherapy. It is possible that patients who received Immunotherapy were taking part in a clinical trial. It is also possible that immunotherapy was recommended in patients who have exhausted many lines of standard-of-care treatments. Furthermore, studies have shown that immunotherapy has been associated with improved survival in microsatellite instability (MSI) positive patients diagnosed with other malignancies [42, 43]. Therefore, patients who have microsatellite instability (MSI) may have a higher chance to receive immunotherapy.

Nonetheless, the findings of the current study are consistent with the results of a few other clinical trials and retrospectives studies. A phase II trial involving 60 patients with resected PDAC, investigated the impact of granulocyte-macrophage colony-stimulating factor (GM-CSF) with chemoradiation reported a median survival of 24.8 months (95% CI, 21.2–31.6) [39]. A dose-escalating study with 24 patients evaluated Gene-mediated cytotoxic immunotherapy (GMCI™) in combination with chemoradiation therapy for resected PDAC in adjuvant setting reported a median OS of 12 months and a 1-year OS of 50% [40]. A multi-institutional open-label phase II study evaluated algenpantucel-L in combination with chemoradiation therapy in 70 patients with resectable PDAC and reported the 12-months OS rate of 86% [41]. In the current study, we found a median OS of 29.31 months, a 12-months OS rate of 88%, and a 24-months OS rate of 60% comparable to these studies.

To our knowledge, the current study is the first to use an extensive database such as NCDB and investigate the impact of immunotherapy on the OS of PAD patients who receive definitive surgery. In this study combining immunotherapy with chemoradiation was associated with significantly improved OS. The results stayed the same when patients who received immunotherapy more than six months before or after chemoradiation were excluded. The findings of our study, together with early findings of some clinical trials, warrant future clinical trials of immunotherapy combined with chemoradiation in PAD patients. Chemotherapy and immunotherapy both induce a systemic immune response, and the addition of RT to chemotherapy and immunotherapy may be required to overcome the local and systemic immune suppression. It will be of particular interest to check the synergic interaction of Immunotherapy with stereotactic body radiation therapy (SBRT) in borderline resectable or locally advanced PC. SBRT is delivered during a short period (1–2 weeks), which will avoid the delay in the start of systemic therapy, including Immunotherapy. Early systemic treatment is essential and is recommended in pancreatic cancer patients to minimize the early spread of the tumor. The synergic interaction between Immunotherapy and SBRT could improve the abscopal response following SBRT. Future studies should focus on investigating the interaction of immunotherapy and SBRT in pancreatic cancer.

The negative results of chemotherapy plus immunotherapy compared to chemotherapy indicates that both systemic and local immune response is necessary to overcome the immune evasion of pancreatic cancer cells. The immunostimulatory effect of chemotherapy, especially in the adjuvant setting is through the inhibition of T regulatory cell and MDSCs rather than the stimulation and increase of T cells [44–46]. The significant improved OS associated with chemoradiation and immunotherapy is biologically justified. Evidence indicates that chemoradiation, especially after surgery, can significantly increase the number and function of dendritic cells by reducing immunosuppressive cytokines [47]. Dendritic cells are an essential part of the immune system and play a critical role in tumor cell recognition and T cells stimulation [48]. Chemoradiation is also capable of producing humoral or cellular immune responses, and its combination with immunotherapy has shown to mount long-term T cell reactivity [49–51].

### Limitations

Despite the large sample size, our research has several limitations. The NCDB does not capture information on performance status, responsiveness to therapy, quality of radiation, the details of staging and follow-up practices, and other unmeasured confounding factors that could bias the analysis. We were also not able to adjust for microsatellite instability (MSI) status, and patients who would have received immunotherapy were likely to have been MSI, which may impact sensitivity to immunotherapy, as well as outcomes. The NCDB database does not provide information about the type of immunotherapy, chemotherapy, and the use of multi-agent chemotherapy regimens. It would have also been exciting to check the impact of Immunotherapy on local, locoregional, and metastases free survival time. Unfortunately, the NCDB does not provide information about local, locoregional, and distant metastases after the treatment initiation. Nonetheless, the NCDB is the largest cancer database in the world which capture the majority of the newly diagnosed cancer cases in the United States and serves as an excellent source outside of multicenter clinical trials for examining the impact of novel treatments such immunotherapy on the OS of PDAC patients who received definitive surgery of the tumor.

## Conclusions

This study is the first large study with a robust analysis using the NCDB that has investigated the impact of immunotherapy in combination with chemotherapy, RT, and chemoradiation on the OS of PDAC patients who received definitive surgery of the pancreatic tumor. In this study, combining chemoradiation therapy with immunotherapy was associated with significantly improved OS of the patients. The findings of the current study, together with the results of other previous studies of the use of immunotherapy with other standard-of-care cancer treatments in PDAC patients who receive surgery, warrant the need for future clinical trials of investigating the impact of immunotherapy in this group of patients.

## Data Availability

The datasets used and analyzed during the current study are available from the corresponding author on reasonable request.

## Abbreviations

NCDB: National Cancer Database
OS: Overall survival
PDAC: Pancreatic adenocarcinoma
MDSCs: Myeloid-derived suppressor cells
RT: Radiation therapy
